# Electro-Therapeutics of Gynecology

**Published:** 1893-03

**Authors:** 


ELECTRO-THERAPEUTICS OF GYNECOLOGY, by Augustin H. Goelet, M. D. In two volumes. Belonging to the Leisure Library Series. Published by Geo. S. Davis, Detroit, Mich.

VOL. I. contains V. chapters devoted to Electro-Physics and Electro-Physiology. It presents a brief resume of the terms used, varieties of batteries currents and their action, and discription of various electrodes used with various accessories for controlling and measuring the currents.

Vol. II. Is devoted to Electro-Therapeutics of Gynecology. It contains V. chapters dealing with the application of electricity in the treatment of disorders of menstruation, diseases of the uterus, diseases of the appendages and broad ligaments, pelvic tumors, nomenclature for records of treatment and list of electrodes necessary for gynecological work.

To those having confidence in electrical treatment of diseases of women, it will prove useful as a practical concise guide in the use of electricity according to latest approved methods.

